# The relationship between perceived social support from family and diabetes self-management among patients in Uganda

**DOI:** 10.11604/pamj.2022.41.279.33723

**Published:** 2022-04-07

**Authors:** Jude Tadeo Onyango, Jane Frances Namatovu, Innocent Kabahena Besigye, Mark Kaddumukasa, Scovia Nalugo Mbalinda

**Affiliations:** 1 Department of Family Medicine, School of Medicine, College of Health Sciences, Makerere University, Kampala, Uganda,; 2 Department of Medicine, School of Medicine, College of Health Sciences, Makerere University, Kampala, Uganda,; 3 Department of Nursing, School of Health Sciences, College of Health Sciences, Makerere University, Kampala, Uganda

**Keywords:** Self-management, perceived social support, diabetes mellitus, self-care, family support, Uganda

## Abstract

**Introduction:**

optimal self-care in diabetes mellitus contributes substantially to good glucose control and delays development of complications. The family´s support is an important predictor of optimal self-care behavior. Little is known about the relationship between social support from family and self-care behavior in Uganda. The study set out to determine the association between perceived social support from immediate family and diabetes self-management among diabetic patients in the eastern region of Uganda.

**Methods:**

this was a cross-sectional study among 405 adults attending diabetic outpatient clinics in Eastern Uganda between May 2021 and June 2021. Data of socio-demographic and clinical characteristics, perceived social support from family, and diabetes self-management were collected. Descriptive statistics were done and associations of socio-demographic and clinical characteristics, perceived social support from family with diabetes self-management were determined using Pearson Chi-square and Fisher´s exact tests.

**Results:**

the mean (SD) age was 52 (14.9) years, (60%) were female, majority (49.4%) were 45-64 years old. Perceived social support from family and optimum diabetes self-management were found in; (95.3%) and (87.4%) respectively. Perceived social support from family was associated with optimal diabetes self-management (p-value <0.001). Financial contribution from family members to cost of care and cohesion among family members in support of care were associated with optimal diabetes self-management both with a (p-value 0.001). Access to a functional glucometer was associated with optimal diabetes self-management (p-value <0.001).

**Conclusion:**

among patients in Eastern Uganda, self-management for diabetes control is significantly associated with perception of receipt of support from their families.

## Introduction

Diabetes mellitus (DM) is a chronic metabolic disorder whose prevalence has increased globally. The rise has been faster in the low- and middle-income countries (LMIC) compared to high-income countries [1,2]. In Uganda, the prevalence of diabetes is reported at 1.4% with a higher prevalence of 7.4% reported in rural Eastern Uganda in 2017 [3-5]. Despite the prevalence of the disease in Uganda and specifically the eastern region where the disease is highly prevalent [4], few studies have examined the relationship between self-management in diabetes and family support [6-10].

The disease invariably has a negative impact on an individual´s quality of life arising from complications when the management of the condition is poor. Diabetes management requires the adoption and sustenance of lifelong health-related behaviours to maintain optimal glycemic levels in order to prevent or delay the incidence of complications responsible for morbidity and mortality associated with the disease [11]. The support of family members is crucial in the sustenance of diabetes self-care behaviour. Although ultimate responsibility remains with the individual, the supportive actions of other members make the daily required tasks to maintain optimum blood sugar levels much easier to accomplish [12-15]. Self-care and better health outcomes are more likely when members bring individual supportive strengths together working as a cohesive functioning family unit [16,17]. Studies around the world have found a positive influence of diabetes-specific supportive behaviour and actions from family members on individuals´ self-management behaviour and health [18-21]. Negative effects have been documented with non-supportive behavior and actions, and non-cohesive and dysfunctional family environments [20,22,23].

Social support from family is especially important to augment professional health care in LMICs including Uganda which has health care systems that face challenges of poorly developed health care infrastructure, insufficient health supplies and patient health workforce ratios [24-26]. The patient´s perception of whether support is provided or is available has been used as an indirect measure of social support from family in a number of studies conducted around the world including Africa [27-30]. Therefore, this study intends to extend the existing evidence to Uganda´s setting by determining the association between perceived social support from family and diabetes self-management behaviour among patients in the eastern region of Uganda.

## Methods

**Study setting and study design:** the study used a quantitative cross-sectional design, carried out between May 2021 and July 2021 at the diabetes outpatient clinics at three public regional referral hospitals located in Eastern Uganda: Jinja, Mbale and Soroti Regional Referral Hospitals. The eastern region is one of the four regions of Uganda. The three hospitals serve as referral hospitals to all the lower-level health facilities in the 32 districts that are located within the geographical region. Each of the hospitals has one day of the week for the diabetic clinic to open and see patients. The hospital medical records show that the number of patients registered with the clinics is 1216, 1515, and 610 for Soroti, Mbale and Jinja, respectively.

**Study population:** the study included: confirmed adult diabetic patients (type I or II) who had been regularly attending the diabetes clinics for at least six months at the three study sites; reported to be living with other people they consider as part of the family and speak any of six languages spoken in the eastern region; English, Lumasaba, Lusoga, Jopadhola, Ateso and Kiswahili. We excluded study participants who were: pregnant, too weak to sustain themselves through the interview process and required admission, and those who had a major psychiatric illness where recognition of perceived family support could be impaired.

**Sample size:** we estimated a total sample size of 405 with a level of significance of 5% at a power of 80% and an anticipated proportion (p1) of diabetic patients with family support who achieve good glycemic control at 60%, with the proportion of diabetic patients with no family support who achieve good glycemic control estimated at 45% and a 10% rate for incomplete information. The proportion from each hospital that made up the total sample size was determined by the ratio of the average patient attendance per clinic day at the three regional referral hospital (RRH)s (120 Jinja RRH: 150 Mbale RRH: 150 Soroti RRH). Therefore, for a total sample size of 405 required for the study, Jinja RRH contributed 105 patients, Mbale RRH contributed 150 patients, and Soroti RRH contributed 150 patients to make up the total sample size.

**Study procedure:** the study participants were selected using a systematic random sampling method at each of the three diabetic clinics. The sampling frame at each clinic was made from the patients expected to come for review and had been registered to be seen on the particular clinic day. The sampling interval at each clinic was determined through dividing the total number of diabetic patients registered with the clinic by the sample size proportion that was to come from the hospital. Since the total number registered per hospital was 1216, 1515, and 610 for Soroti, Mbale and Jinja respectively, a sampling interval of 12 was used at two hospitals, Soroti and Mbale RRHs and 6 was used for Jinja RRH. A random start number (patient) was determined using simple random sampling and then every twelfth patient (Soroti and Mbale RRHs) and sixth patient (Jinja RRH) thereafter was approached and asked if they could participate in the study. If the patient objected then the next person registered would be approached to participate.

A trained research assistant explained to the potential study participants the purpose of the study and checked for inclusion criteria that included reviewing the patient´s medical records. Those who met the criteria were then requested to participate in the study. Family members who had accompanied the patient were requested to leave the study area to allow the participant to complete the study. Each participant was interviewed once in the pre-consultation sitting area before the consultation. All study participants provided written informed consent before any study procedures were started.

**Study variables and data collection tool:** the data were collected using a three-part structured interviewer-administered questionnaire. Social demographic and clinical characteristics: thirty-five items, including among others: age, sex, level of formal education completed, marital status, duration of the marriage, number of people in the household, number of people in the household that are employed, living arrangement, duration of diabetes diagnosis, use of insulin for treatment of diabetes, access to a glucometer, receipt of formal structured self-management education by the patient and family members, and documentation for guidance on self-management at home. The diabetes type was obtained from medical records. Self-care activities: assessed using the diabetes self-management questionnaire (DSMQ) scale [31]. It has sixteen items that assess specific diabetes self-management activities that predict glycemic control including: adherence to diabetes diet, physical activity, self-blood glucose monitoring, medication adherence and use of health care follow up appointments.

**Social support from family:** assessed using the perceived social support from family (PSS-Fa) scale. It consists of twenty items that assess the patient´s level of perceived family support. It examines how the patient perceives support, information and response from their family. The DSMQ and the PSS-fa scales have been used in other African settings to assess diabetes self-management [30,32] and social support from family [30,33]. Pre-testing of the questionnaire was done among twenty diabetic inpatients at each of the three study sites, and editing was done to ensure the questions were appropriately structured for uniform and correct understanding. The following were eventually taken as negatively worded statements in the PSS-Fa and the DSMQ scales: statements III, IV, XVI, XX, XIX and statement 5, 7, 10, 11, 12, 13, 15, 16 for the PSS-fa and DSMQ scales respectively. Statement 13 of DSMQ was better understood as “huge amounts of food” instead of “food binges” and was modified as such. Statement 14 was generally understood as a positively worded statement and was taken as such. The interview took about 30 minutes then each questionnaire was checked for completeness.

**Data entry:** data entry was done using EpiData entry software version 3.1, cleaned, coded, and then exported to STATA statistical package version 15 for data analysis.

**Operational definitions:** the PSS-Fa scale scores were categorized as ‘strong perceived family support´ if score is above or equal to 11, 'weak perceived family support' if score is (7-10), and 'no perceived family support' if the PSS-Fa score is below or equal to 6. For testing the hypothesis, recategorization was done for perceived social support from family - strong and weak (score 7 and above), and no perceived family support (6 and below). The transformed DSMQ sub scale and sum scale scores were categorized as follows: ≤6/10 for sub optimal self-management and >6/10 for optimal self-management.

**Data analysis:** tests for normality were performed using the Shapiro-Wilk test. Descriptive statistics were used to describe basic features of collected data. Continuous variables were summarized using the mean (SD) and median (IQR) for normally distributed and non-normally distributed data. The categorical variables were summarized using frequencies (percentages). Correlation coefficients were determined between perceived social support from family-scale score (PSS-Fa scale) and DSMQ sum and sub scales. Tests were done to determine if there was a statistical difference between the data from the three hospitals. (ANOVA-parametric, KRUKAL-WALLIS-non-parametric). Pearson Chi-square test was used to determine the association between perceived social support from family and diabetes self-management (DSM). The associations of socio-demographic and clinical characteristics and diabetes self-management were also determined using Pearson Chi and Fisher´s exact tests were necessary. The associations were considered statistically significant if the p value was ≤0.05.

**Ethical approval and consent to participate:** Research and Ethics Committee (REC) of the School of Medicine (SOM), Makerere University College of Health Sciences (MakCHS) approved the study (#REC REF 2020-142). Written informed consent was obtained from all participants.

## Results

The socio-demographic characteristics of the study participants are presented in Table 1. A total of 405 adults with diabetes participated. The mean age of the participants was 52 (SD 14.9) completed years and females made up the majority of the sample (60%) with males at (40%). Most of the participants were: Christian (79.3%), married or cohabiting (67%), and had at least completed primary education (78.3%). A big majority originated (89.6%) and almost all (99%) resided within the country´s eastern region of the country with a sample median distance of 10 km from the hospital. With regard to employment, (81.2%) had some form of employment and the median monthly income of the participants was (28 (8,56)) USD. The median number of people in the family who were employed was (1 (0,2)). More than half (55.9%) were living in extended families with a sample median house hold size of 7 people. Nearly all participants received some form of domestic help related to managing their diabetes condition and over half reported some or high cohesion among family members. However, the majority (64.2%) reported minimal or no financial contribution from family to the cost related to hospital care.

**Table 1:** bivariate analysis of socio-demographic characteristics and diabetes self-management

	Suboptimal self-mgt (≤ 6)	Optimal self-mgt (> 6)	Overall	
Parameter	(N=51)	(N=354)	(N=405)	p-value
**Sex**				1.000**
Male	20 (12.3%)	142 (87.7%)	162 (40.0%)	
Female	31 (12.8%)	212 (87.2%)	243 (60.0%)	
Age (completed years)	49 (17.14)	51 (14.57)	51 (14.91)	0.384μ
**Marital status**				0.258**
Married/cohabiting	29 (10.7%)	241 (89.3%)	270 (67.0%)	
Separated/divorced	16 (16.5%)	81 (83.5%)	97 (24.1%)	
Widowed/widower				
Single/never married	6 (16.7%)	30 (83.3%)	36 (8.9%)	
**Highest level of formal education (completed)**				0.861*
None	10 (11.4%)	78 (88.6%)	88 (21.7%)	
Primary	23 (13.6%)	146 (86.4%)	169 (41.7%)	
Secondary	14 (13.6%)	89 (86.4%)	103 (25.4%)	
Tertiary/university	4 (8.9%)	41 (91.1%)	45 (11.1%)	
Distance from place of residence	15 (8, 24)	10 (5, 20)	10 (5, 20)	0.026 β
**Employment status**				0.395*
Formally employed	1 (3.8%)	25 (96.2%)	26 (6.4%)	
Informal/self-employed/peasant	39 (12.9%)	264 (87.1%)	303 (74.8%)	
Unemployed	11 (14.5%)	65 (85.5%)	76 (18.8%)	
Income per month (USD)	19 (6, 56)	28 (10, 69)	28 (8, 56)	0.105β
House hold size	8 (5, 11)	7 (5, 10)	7 (5, 10)	0.953β
No. employed in the home	1 (0, 2)	1 (0, 2)	1 (0, 2)	0.072β
**Living arrangement with other members of family**				0.125*
Nuclear family only	19 (18.4%)	84 (81.6%)	103 (25.9%)	
Extended family	24 (10.8%)	198 (89.2%)	222 (55.9%)	
Polygamous family	2 (5.3%)	36 (94.7%)	38 (9.6%)	
Single parent family	5 (14.7%)	29 (85.3%)	34 (8.6%)	
**Domestic help from family in managing diabetes at home**				0.429*
Yes	2 (18.2%)	9 (81.8%)	11 (2.7%)	
No	49 (12.4%)	345 (87.6%)	394 (97.3%)	
**Financial contribution from family to cost of care**				0.001*
No/non	15 (27.8%)	39 (72.2%)	54 (13.3%)	
Minimal	27 (13.1%)	179 (86.9%)	206 (50.9%)	
Most	5 (6.1%)	77 (93.9%)	82 (20.2%)	
All	4 (6.3%)	59 (93.7%)	63 (15.6%)	
**Cohesion among family**				0.001**
No/non	5 (26.3%)	14 (73.7%)	19 (4.7%)	
Minimal	29 (19.3%)	121 (80.7%)	150 (37.0%)	
Some amount	6 (7.8%)	71 (92.2%)	77 (19.0%)	
Highly cohesive	11 (6.9%)	148 (93.1%)	159 (39.3%)	

** Pearson Chi-square test; μ: independent sample t-test; * Fisher's exact Chi-test; β Wilcoxon rank-sum test

Table 1 also shows details of the association of socio-demographic characteristics with self-management. Only, distance from residence to hospital, financial contribution by family members to cost related to hospital care, and cohesion among family members were observed to have a statistically significant association with self-management. Significantly, a lower distance away from the hospital, more financial contribution, and cohesion among family members was found among those with optimal self-management. Participant´s sex, age, marital status, education level completed, monthly income, living arrangement at home, number of people at home, number of family members employed, and having domestic help to manage the condition, were not associated with self-management.

Table 2 shows the association of clinical characteristics with diabetes self-management. The type of diabetes, duration with and on diabetes treatment, access to a functional glucometer, monthly hospital visits, and having been hospitalized in the last three months, were significantly associated with self-management. Having type II diabetes, lower duration with diabetes or diabetes treatment, having a functioning glucometer, visiting the hospital at least monthly, and not having been hospitalized for diabetes within the last three months were observed to be statistically associated with optimal self-management. There was no statistically significant difference observed about whether a participant had other chronic disease/s, whether a participant or their family members had received diabetes and its management (DSME), or whether there was use of documents for guidance and/or reference in managing diabetes at home.

**Table 2:** bivariate analysis of clinical characteristics and diabetes self-management

	Suboptimal self-mgt (≤ 6)	Optimal self-mgt (> 6)	Overall	
Parameter	(N=51)	(N=354)	(N=405)	p-value
**The type of diabetes (documented)**				0.021**
Type I DM	8 (25.8%)	23 (74.2%)	31 (7.7%)	
Type II DM	43 (11.5%)	331 (88.5%)	374 (92.3%)	
Duration with diabetes since diagnosis (years)	6 (3, 11)	4 (2, 9)	5 (2, 10)	0.037β
Duration on drugs/treatment	6 (3, 11)	4 (2, 9)	4 (2, 9)	0.038β
**Access to a functional glucometer**				<0.001*
Own one	1 (2.1%)	47 (97.9%)	48 (11.9%)	
Don't own but can access one	26 (10.3%)	226 (89.7%)	252 (62.2%)	
Can't access one	24 (22.9%)	81 (77.1%)	105 (25.9%)	
**Ever received diabetes self-management education (DSME)**				0.086*
Yes	47 (12.0%)	344 (88.0%)	391 (96.5%)	
No	4 (28.6%)	10 (71.4%)	14 (3.5%)	
**Family member/s ever had diabetes self-management education (DSME)**				0.208**
Yes	10 (9.2%)	99 (90.8%)	109 (26.9%)	
No	41 (13.9%)	255 (86.1%)	296 (73.1%)	
**Have/use any documentation to guide or refer to in managing diabetes**				0.161**
Yes	7 (8.1%)	79 (91.9%)	86 (21.2%)	
No	44 (13.8%)	275 (86.2%)	319 (78.8%)	
**Has other chronic diseases apart from diabetes**				0.238**
Yes	22 (10.7%)	184 (89.3%)	206 (50.9%)	
No	29 (14.6%)	170 (85.4%)	199 (49.1%)	
**Frequency of hospital visits in the last three months**				0.053**
≤Monthly	35 (10.8%)	289 (89.2%)	324 (80.2%)	
Beyond a month	15 (18.8%)	65 (81.3%)	80 (19.8%)	
**Ever been hospitalized because diabetes in the last three months**				<0.001**
No	36 (10.1%)	321 (89.9%)	357 (88.1%)	
Yes	15 (31.3%)	33 (68.8%)	48 (11.9%)	

** Pearson Chi-square test; μ independent sample t-test; * Fisher's exact Chi-test; β Wilcoxon rank-sum test

The association details of perceived social support from family with diabetes self-management and fasting blood glucose levels are presented in Table 3. The study found a statistical association with diabetes self-management (p<0.001). The proportion of those with optimal self-management who had family support was significantly different from the proportion of those with suboptimal self-management who had family support. Table 4 shows a significant positive correlation of PSS-fa with; glucose management, dietary control and physical activity subscales, and overall DSMQ sum scale.

**Table 3:** bivariate analysis of perceived social support from family and diabetes self-management

	Suboptimal self-care (≤ 6)	Optimal self-care (> 6)	Total	
	(N = 51)	(N = 354)	(N = 405)	p-value
**Perceived social support from family - PSS-Fa scale**				<0.001**
**≤**6, no support	7 (13.7%)	12 (3.4%)	19 (4.7%)	
7-10, weak	8 (15.7%)	19 (5.4%)	27 (6.7%)	
>11, strong	36 (70.6%)	323 (91.2%)	359 (88.6%)	

** Pearson Chi-square test

**Table 4:** correlation between perception of support and diabetes self-management (N=405)

Parameter	Median (IQR)	Correlation	p-value
Glucose management sub scale	7 (6,8)	0.148	0.0029
Dietary control sub scale	7 (6,8)	0.098	0.0497
Physical activity sub scale	8 (7,10)	0.120	0.0157
Health care use sub scale	10 (9,10)	0.018	0.7132
DSMQ sum scale	8 (7,8)	0.165	0.0008

## Discussion

Social support from family is crucial for improving and sustaining self-management practices for people suffering from chronic diseases, including diabetes. It is an established fact that most of the management of diabetes takes place within the context of family. The existing literature shows that diabetic patients who are better supported and feel supported by people in their immediate context get to perform better at self-care practices that are required for their glycemic control. This is the first study to determine the association of perceived social support from family with self-management practices in Uganda. In low-income countries, there is limited social protection supports for health care, this is then shouldered by the immediate families who are a key resource for care and support (reference). In this study, majority of the study participants had social support from their immediate families and had a strong perception of social support. This is similar in other studies done in Nigeria [30,33], Burkina Faso [34] which have comparable family and cultural settings, and Iran [35].

The Ojewale *et al*. study used the mean of the group´s responses as the cut off for “good” versus “poor” PSS-fa; the Osuji *et al*.study found PSS-fa at 84.7%, the Traoré S *et al*.study found family support at 85.19%, which compare with this study´s finding of 95.3% (strong and weak PSS-fa), the Majlessi *et al*. study did not categorize PSS-fa but the mean was 13.13 (SD1.8) and 12.89 (SD2.56) for type 1 and type 2 respectively which are both above ≥6 the cut off for “no PSS-fa” used for this study. However, overall, the three studies show the presence of family support for family members who have diabetes in the settings mentioned.

In this study, financial contribution from family was mostly minimal. The explanation for this could be found in the generally low economic status (mean monthly of 50.1 USD) of the majority participants considering the prevailing costs that were to be incurred in care for: food, transport to hospital, and all or part of their medication among other needs. The mean number of people in the home who had some form of employment was 1.5 (1.32) which means that there were not many other people in the home who could make a financial contribution. In addition, these people could be of a similar economic level. This situation is made worse when there are financial needs from other people to be catered for as shown by the relatively large house hold size (mean of 8.1 SD 4.51), the majority living in an extended or polygamous family arrangement. It has been found that low income puts limitations on the implementation of self-management practices [36,37].

This study also found that participants scored highly on the diabetes self-management scale (mean transformed score of the group was 7.47 SD 1.21). This is consistent with the study by Ojewale *et al*. which found 61.9% of the study participants had a “good DSMQ scale” score. The cut off for optimal self-management was >6 on the transformed score compared to the study by Ojewale *et al*. where the cut off for good self-management was based on a score of the mean or above of the group´s scores. The good scores on diabetic self-management in the two studies could, among other factors, be attributed to the social support they receive from family members. The evidence in the literature shows that diabetic patients are more likely to do better at self-management when they feel supported by the important others in their social cultural context and the family are the primary providers of social support [29,38,39]. This study also shows a significant association of perceived social support from family with self-management of diabetes (p-value <0.001). This is also shown by the significant positive linear correlation between perceived social support from family (PSS-fa) and three subscales and the sum scale of the diabetes self-management (DSMQ scale). A positive correlation with health care use was observed although this was not statistically significant.

This study also found other socio-demographic variables that were significantly associated with self-management. Longer distances from residences to hospital (p-value 0.026) usually coupled with poor road terrain and inadequate means of transport to reach the hospitals are demotivators for seeking routine, urgent or emergency health reviews. This concurs with one Ghanian study that found among others, long-distance to hospital as a barrier to diabetes self-care [40]. Another in Kenya found that distance impacts on frequency of clinic attendance [41]. In this study, a big majority are provided with financial assistance (86.7%) to meet the bills for transport and medicines that were prescribed but often not in stock at the hospitals. We see that having financial assistance from family was significantly associated with self-management aspects such as regular medication intake and health care reviews (p-value 0.001). In the context of low personal incomes and insufficient medical supplies in public hospitals, patients have no alternative but to rely on family members for support. This is not unique to this study. A similar finding where many patients have to rely on family members for financial support has been reported in a systematic review by Suglo JN and Evans C [42].

Cohesion among family members in support of the patient´s care needs was significantly associated with self-management in this study (p-value 0.001). Studies by Burnet *et al*. and Bennich *et al*. provide evidence of the impact of collaborative and supportive interactions of family members on patient´s self-efficacy and diabetes self-care among other important variables [21,43]. One important clinically related variable was the inability to access a functioning glucometer to measure and monitor their blood glucose levels out of the hospital setting which was noted in (25.9%) of participants and only (11.9%) owned one. This study observed a significant association between having access to a functioning glucometer and reporting optimal self-management (p-value <0.001). The proportions of those with optimal self-management among those who could not access a functional glucometer was significantly lower than the proportion of those with optimal self-management among those who could access a functional glucometer. The possibility of measuring one´s glucose levels is a motivating factor for self-monitoring of glycemia status. The absence of or inaccessibility to a functional glucometer and/or glucose measuring strips creates a significant challenge to diabetes management both at home and at the health facilities in the region where availability is not always consistent. Studies by Mogre V *et al*. [40,44] and Wolderufael M and Dereje N conducted in Ghana an Ethiopia respectively found lack of a glucometer as a barrier to diabetes self-care.

Diabetes and its management (DSME) is known to have a positive effect on self-management [45]. This effect is more likely to be sustained when other family members acquire knowledge about the disease and the skills to perform self-management tasks. They are more likely to be of meaningful and practical help to the family member who has diabetes [46]. Nearly all patients (96.5%) and (26.9%) of family members in this study had gotten information about diabetes and its management (DSME) respectively. This could be explained by the existing practice at each hospital of regular health education sessions for diabetic patients receiving care at the clinics enabling most patients to acquire knowledge about the disease and requirements for its management. This could also be a contributory factor to the finding of the majority of participants in this study reporting optimal self-management (mean score of 7.47). Though this study did not find a significant association of DSME of family members with patient self-management, family members are likely to be more supportive when they are informed and equipped with skills to help affected members manage their condition [47]. The proportion of those with suboptimal self-management in type I diabetics was significantly higher than that among type II diabetics (p-value 0.021). This result can be explained by the additional challenges in treating type I diabetes requiring the use of injectable insulin and the fact that patients with type I are younger compared to those with type II disease. In this study, longer duration with the disease and being on treatment was associated with reporting suboptimal self-management (p-values of 0.037 and 0.038, respectively). This could be due to the daily stressful diabetes management demands that the disease puts on sufferers. In this study, we also found that the proportion reporting suboptimal self-management among those who had been hospitalized due to diabetes within the last three months was significantly higher than that among those who had not been hospitalized. This is explained by the established fact that poor self-care practices for chronic diseases like diabetes make more likely the development of severe disease that necessitates hospital admission.

**Study strengths and limitations:** this is the only study of its kind in Uganda that appraises the association of family social support with diabetes self-management. Measurements: the DSMQ scale has shown a high correlation with glycemic control compared to other self-care instruments during it is development and evaluation and during its use in other studies. However, DSMQ scale has been validated and used in other settings but has never been validated among diabetic patients in Uganda. The scale was initially developed to be self-administered. In this study, the scale was interviewer-administered. This could have introduced bias. The study was conducted at all the three regional hospitals which are the highest-level referral health facilities in the region making the findings more likely to be representative of the situation Eastern Region of Uganda. However, the generalizability of the findings could be limited due to the fact that there are patients who do not receive care at the three regional hospitals and do not speak any of the six languages selected for this study. Also, the majority of patients who visit public hospitals are of low socio-economic status and therefore might not represent other patient groups. Recall bias could not be entirely excluded because the study involved the recall of past events and circumstances. A causal relationship could not be determined. The potential for reverse causality could not be excluded. The fact that the study did not involve inpatients could have excluded important information on the relationship between family support and self-management.

## Conclusion

Perceived social support from family was significantly associated with diabetes self-management. It is important to design family involvement community programs to target family member diabetes education. These programs could create expert patient groups to help patients cope with the stressful demands of the disease and encourage cohesion and active participation among family members in domestic activities, mobilization of financial resources in support of self-care practices that enable affected members achieve optimal glycemic control. Provision of consistent access to a functional glucometer is a necessary prerequisite for every diabetic at home or at nearby health facilities that can be easily accessed. This can be one effective strategy to motivate diabetic patients and their families to engage in self-care practices to avoid unnecessary complications and hospitalization among the affected members.

### What is known about this topic

- Social support from family impacts positively on diabetes self-care behaviour of affected members;
- Lack of social support and negative behaviour/actions from family members impacts negatively on diabetes self-care behaviour of affected members.

### What this study adds

- This study´s findings extend this existing evidence to Uganda´s health care setting;
- The opportunity to do further research to examine: the relationship of family support with glycemic control, factors associated/reasons for perceived social support from family and diabetes self-care behaviour and diabetic patients, and to validate the PSS-fa and DSMQ research tools.
